# Initial Responses of Articular Tissues in a Murine High-Fat Diet-Induced Osteoarthritis Model: Pivotal Role of the IPFP as a Cytokine Fountain

**DOI:** 10.1371/journal.pone.0060706

**Published:** 2013-04-12

**Authors:** Munetaka Iwata, Hiroki Ochi, Yasushi Hara, Masahiro Tagawa, Daisuke Koga, Atsushi Okawa, Yoshinori Asou

**Affiliations:** 1 Division of Veterinary Surgery, School of Veterinary Medicine, Nippon Veterinary and Life Science University, Musashino-shi, Tokyo Japan; 2 Laboratory of Veterinary Microbiology, Nippon Veterinary and Life Science University, Musashino-shi, Tokyo Japan; 3 Department of Orthopaedic Surgery, Tokyo Medical and Dental University, Bunkyo-ku, Tokyo Japan; McGill University, Canada

## Abstract

Obesity and high body mass index are associated with a higher incidence of osteoarthritis (OA). The aim of this study is to investigate the involvement of the infrapatellar fat pad (IPFP) in the sub-acute effect of a high fat diet (HFD) on the development of knee-OA. C57BL/6J male mice were fed either a HFD or a normal diet beginning at seven weeks of age. Tissue sections were evaluated with immunohistological analysis. The IPFP was excised, and mRNA expression profiles were compared using real-time RT-PCR analysis. Osteoarthritic changes were initiated in the HFD group after eight weeks of the HFD. Increased synovial cell number and angiogenesis at the anterior edge of the tibial plateau were exhibited prior to osteophyte formation. Quantitative histological analysis indicated that osteophyte volume was significantly increased in the HFD group after eight weeks, along with an increase in the IPFP volume, the size of individual adipocytes and the number of vessels in the IPFP. Histomorphometrical analysis revealed osteophyte area was significantly associated with IPFP area, individual adipocyte area and vascular area. Real-time RT-PCR analysis demonstrated elevated mRNA expression of inflammatory cytokines, growth factor, and adipokines in the IPFP after eight weeks of the HFD. These findings are in parallel with increased expression of the CD68 macrophage marker after eight weeks of the HFD. Expression levels of the adipokines were significantly correlated with expression of TNF-α, VEGF and TGF-β. Immunohistological analysis revealed that the Nampt protein was highly expressed in the IPFP especially around the site of osteophyte formation. Apoptosis and proliferation of chondrocytes were both enhanced at the site of osteophyte formation, indicating higher cell turnover at this region. These observations suggest the IPFP plays a pivotal role in the formation of osteophytes and functions as a secretory organ in response to a HFD.

## Introduction

Osteoarthritis (OA) is a chronic degenerative joint disorder characterized by articular cartilage destruction and osteophyte formation, and is prevalent in society as a major cause of disability. OA risk factors identified by previous epidemiologic studies are limited to age, trauma history, occupation, gender and obesity [1]. Obesity and high body mass index are associated with a higher incidence of OA [2]–[5].

Excess weight caused by obesity introduces increased weight bearing on the knee joints, implicating the influence of mechanical factors in the development of OA especially in major joints of the lower extremity [6]. Trauma, joint instability, and developmental dysplasias are also recognized as predisposing factors in animal models of OA [1]. A number of cohort studies have demonstrated that obesity is an independent risk factor for hand OA [7], [8]; however, mechanical stress cannot explain such a correlation. Therefore, it has been hypothesized that one or more systemic factors are responsible for the correlation between obesity and OA.

Obesity is also associated with an increased amount of adipose tissue, which is recognized as having potent endocrine activity and can give rise to inflammation. Adipose tissue expresses and secretes a variety of bioactive peptides, known as adipokines, which act at both the local (autocrine/paracrine) and systemic (endocrine) level [9].

Activation of adipose tissue macrophages within fat depots is also accompanied with the development of an obesity-induced proinflammatory state [10], [11]. Chronic inflammation triggered by obesity is associated with several diseases such as type 2 diabetes, defective immunity, hypertension, atherosclerosis and several cancers [12]. These studies suggest that inflammatory molecules secreted from adipose tissue may provide a critical connection between obesity and OA.

The infrapatellar fat pad (IPFP) is located in the knee underneath the patella, between the patellar tendon, femoral condyle and tibial plateau, and is positioned closely to the synovial layers and cartilage surfaces of the knee joint. The IPFP contains adipocytes and has an increased number of immune cells such as lymphocytes, monocytes and granulocytes that have migrated from the blood circulatio [13]. IPFPs from OA patients contain inflammatory cytokines, such as basic fibroblast growth factor (bFGF), vascular endothelial growth factor (VEGF), tumor necrosis factor (TNF) alpha, and interleukin (IL) 6 [14]. Thus, the IPFP could play an important role in the initiation and progression of knee-OA. However, the precise roles of the IPFP at the initiation of OA, for instance, or whether a HFD initiates the IPFP to produce more inflammatory mediators, has not been elucidated.

Activation of the synovial layer (synovitis) is seen in many osteoarthritic joints and formation of osteophytes at the junction of the periosteum and synovium is a common feature [2] [3]. This process is initiated with the formation of chondrophytes, followed by chondrocyte hypertrophy. Finally, chondrophytes develop into osteophytes as a result of ossification. However, it is unknown what initiates these processes.

Various laboratories have established in vivo OA models in order to study the mechanisms of OA development. [1], [15]–[18] [19]–[21] Providing a HFD has been shown to increase the incidence of OA in male mice of C57Bl6 strain [19], [20]. In order to investigate the mechanisms of OA initiation, the initial reaction of the knee joints in response to a HFD was evaluated. A detailed histological investigation has been employed to permit rapid evaluation of the murine knee joints as a consequence of a HFD. Histological grading analyses for assessment of OA were utilized rather than quantitative analyses, as there is a lack of measurable markers for OA.

Taken together, we hypothesized inflammatory responses would occur in the IPFP in advance of the initiation of OA. To clarify the role of the IPFP, we induced OA with a HFD and investigated the initial responses of the knee articular cartilage and the IPFP by detailed histological analysis and real-time RT-PCR analysis.

## Materials and Methods

Male C57BL/6J mice were purchased from Sankyo Labo (Tokyo, Japan). Ethical approval was obtained from the institutional review board of Tokyo Medical and Dental University. C57Bl6J mice were fed a diet containing 32% fat for the HFD group or 4.8% fat for the control group (HFD32 and CE-2; CLEA Japan, Inc. Tokyo, Japan) [22] from the age of seven weeks. All of the animals were allowed unrestricted activity and were provided food and water *ad libitum*. None of the mice died during the experimental period.

### Assessment of OA Severity

**Figure 1:**
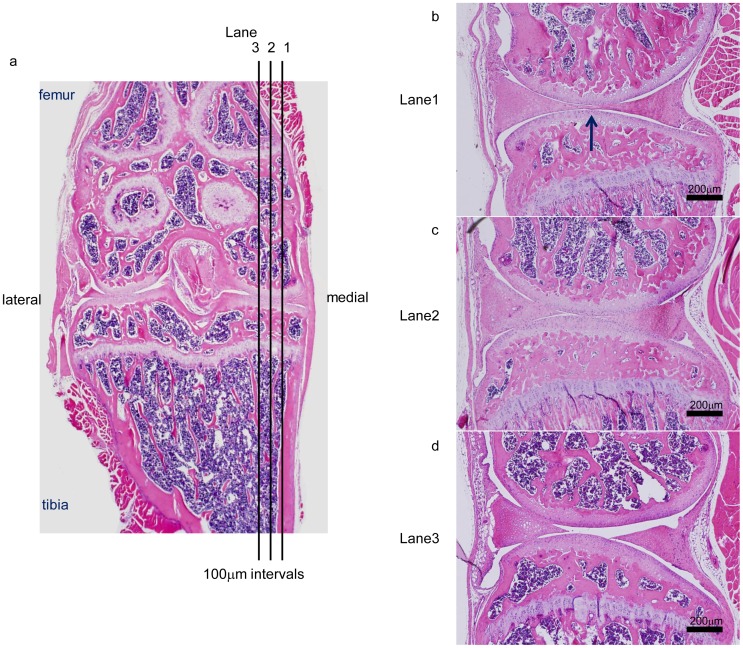
Definition of Lanes 1, 2 and 3. Knee joints were sliced along the sagittal axis (a). Lane 1 is the section in which the central region of the medial meniscus was continuous (b, arrow). Lane 2 and Lane 3 are 100 µm and 200 µm lateral to Lane 1, respectively (c, d).

**Figure 2:**
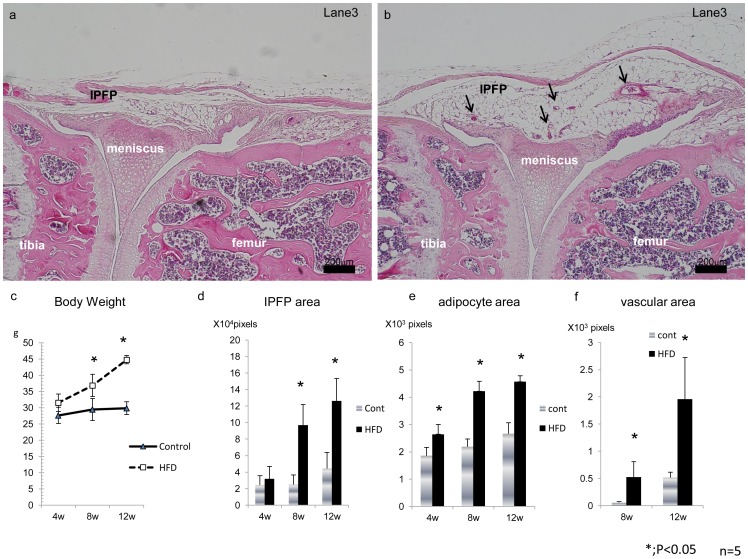
Histological analysis of the IPFP of mice fed a HFD and a normal diet. Sections of articular cartilage from control (a) and HFD (b) mice stained with HE. Note advanced angiogenesis (arrows) and hypertrophic adipocytes in (b). (c); HFD mice weighed 10% more than normal diet mice by four weeks, 20% more by eight weeks and 50% more by twelve weeks. (d–f); Histological quantification of the IPFP area (d), average individual adipocyte area (e) and total vascular area (f) in IPFP. * = P<0.05 using the Mann-Whitney U test.

Mice were sacrificed at four, eight, and twelve weeks after initiating the diet (n = 10 at each time point). Whole knee joints were removed by dissection, fixed in 4% paraformaldehyde, and decalcified in EDTA. After dehydration and paraffin embedding, serial 5-µm sagittal sections were made from the whole medial compartment of the joint. Three sections (from lanes 1–3, Figure 1a, b) were obtained at 100-µm intervals from the weight-bearing region of each knee joint. Lane1 was defined as the section in which the central region of the medial meniscus was continuous (Figure 1b, arrow). Lane2 and Lane3 were 100 µm and 200 µm lateral to Lane1 respectively. The sections were stained with Safranin O–fast green or HE. OA severity in the tibial plateau was evaluated according to a cartilage destruction score (1). Quantitative osteophyte determination was made in the sections from lane 1 (Figure 1a, b) using Image-Pro Plus 4.1 software (Media Cybernetics, Carlsbad, CA). The protruded region, which stained green by Safranin-O staining (Figure 2g–i), was defined as bony osteophyte and quantified.

### Histomorphometry Analysis

Histomorphometric measurements were performed using image analysis software (Image Pro Plus 4.1, Media Cybernetics, Carlsbad, CA, USA). Based on immunostaining for anti-CD31 antibody, immunopositive cells were defined as endothelial cells and the vascular areas were quantified. For quantification of adipocytes, over 30 adipocytes in IPFP were selected and the area of each cell was quantified and averaged.

### RNA Extraction and Real-time RT-PCR

The IPFP tissue was excised using a surgical microscope and microsurgical technique at the previously indicated periods. Total RNA was extracted from the IPFP using TRIzol according to the manufacturer’s directions (Invitrogen). Real-time RT-PCR was performed using the SuperScript III Platinum Two-Step qRT-PCR kit with SYBR Green on the Mx3000P® QPCR System. Briefly, 0.5 µg total RNA was incubated with 10 µl 2× RT reaction mix and 2 µl RT, and then incubated for 50 min at 42°C. The reaction was terminated by incubating for 5 min at 85°C. The cDNA mixture was then incubated for 30 min at 37°C in the presence of RNase H. The PCR reaction was carried out using a mixture of Platinum SYBR Green qRT-PCR Super-Mix UDG, the template cDNA, 10 mM of the primer mix, and DNase-free H_2_O with a total volume of 20 µl per well. The cycling conditions were performed as indicated in the Invitrogen SuperScript™ III Platinum two-step qRT-PCR kit with SYBR Green. Gene expression was normalized to the endogenous control GAPDH, and fold changes in the genes of interest were determined using the comparative threshold cycle (Ct) method.

### Immunohistochemistry

The protein expression of CD31, Nampt, PCNA or TGF-β1 was examined by immunohistochemistry with anti-mouse CD31 antibody, anti-mouse Nampt antibody, anti-mouse PCNA antibody (Abcam Biochemicals, Cambridge, UK), or anti-mouse TGF-β1 antibody (Santa Cruz Biotechnology, INC.) used according to the manufacturer’s instructions. Briefly, tissue sections were incubated overnight at 4°C with primary antibodies, followed by a 30-min incubation at room temperature with appropriate secondary antibodies. Next, the signal was visualized using peroxidase-conjugated avidin and diaminobenzidine from a Vectastain kit, according to the manufacturer’s instructions (Vector Laboratories, Burlingame, CA).

### TUNEL Assay

The TUNEL assay was performed using a TUNEL detection kit according to the manufacturer's instructions (Takara Shuzo, Kyoto, Japan). A section was procured from lane 2 (Figure 1a, c) of each specimen and incubated with 15 µg/ml of proteinase K for 15 min at room temperature, then washed with Phosphate Buffered Saline (PBS). Endogenous peroxidase was inactivated with 3% H_2_O_2_ for 5 min at room temperature. After washing with PBS, sections were immersed in buffer containing deoxynucleotidyl transferase and biotinylated dUTP and incubated for 90 min at 37°C in a humid atmosphere. After washing in PBS, signals were examined by fluorescence microscopy and the number of TUNEL-positive cells in the articular cartilage above the tidemark was determined.

### Statistical Analysis

Data are expressed as the mean ±1 SD. Statistical analysis was performed with the Mann-Whitney U test. P values less than 0.05 were considered significant. Pearson linear regression was used to determine the degree of association between mRNA expression of adipokine or inflammatory cytokine and histological values. The linear regression coefficient R^2^ were reported. Values of P<0.05 were accepted as significant.

## Results

### Impact of HFD on IPFP Histology Over Time

Mice fed the HFD weighed 10% more than normal diet mice by four weeks, 20% more by eight weeks and 50% more by twelve weeks (Figure 2c, p>0.05). Histological examinations of the slides from Lane 3 were made to estimate the effect of the HFD on IPFP histology (Figure 2a, b). The total volume of the IPFP was increased in the HFD group (Figure 2b, d) when compared to the normal diet group (Figure 2a). Average individual adipocytes found in the IPFP were also significantly increased from week eight of the HFD diet when compared to the control group mice (Figure 2e). Concurrently, active angiogenesis was observed in the IPFP of the HFD group (Figure 2f).

**Figure 3:**
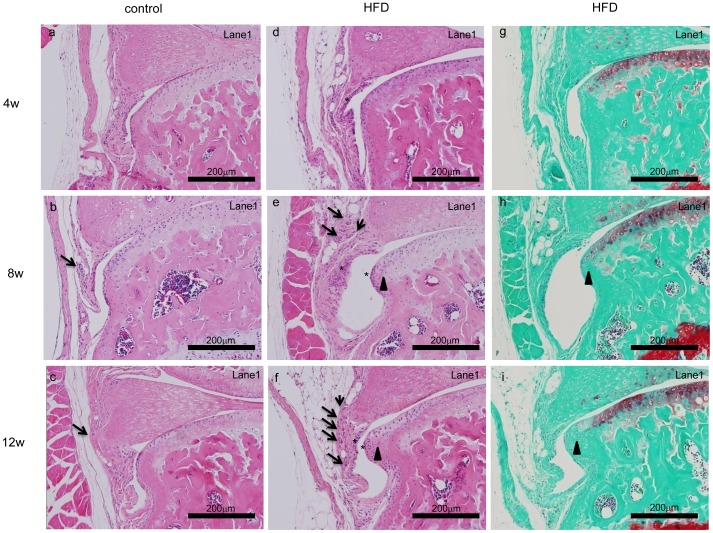
High magnification of the anterior edge of the tibial plateau in the HFD and the control diet mice at the indicated weeks after diet initiation. Representative HE stained sections (a–f) from control mice (a–c) and mice fed the HFD diet (d–f). g, h, i are neighbor sections in Safranin-O staining of d, e, f respectively. Increased synovial cell aggregation was observed at the anterior edge of the tibial plateau and synovium in the HFD (asterisk) mice beginning at week eight. Simultaneously, angiogenesis was activated within the IPFP (arrows) in the HFD group. Osteophytes were first noted at week eight of the HFD (triangles).

**Figure 4:**
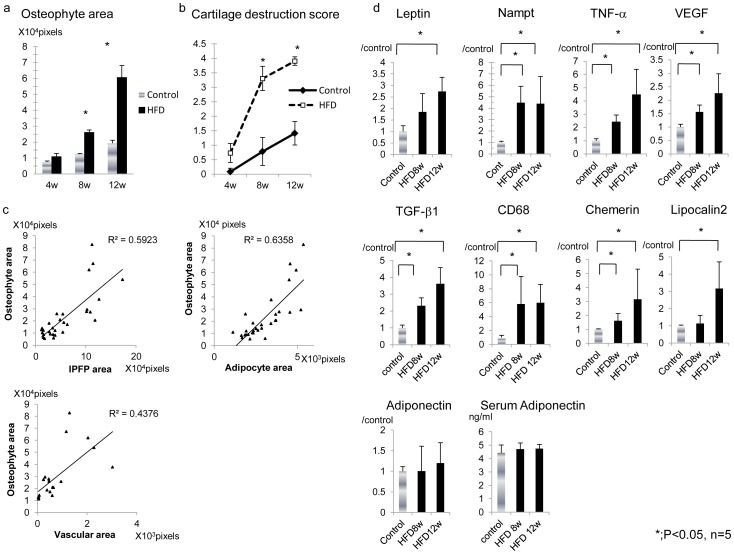
Quantification of osteophytes and real-time RT-PCR analysis of the IPFP. a; The mean osteophyte volume in the control and the HFD group at indicated weeks after onset of the diet. Osteophyte volume was significantly increased in HFD mice beginning at week eight. Reported values are the mean ±1 standard deviation (SD). b; Histological changes in the OA joints were assigned cartilage destruction scores. Values reported include the mean ±1 SD of five to ten mice per group. * = P<0.05 by Mann-Whitney U test. (c–h); mRNA expression of IPFP Leptin, Nampt, TNF-α, VEGF, TGF-β1 and CD68 for both the HFD and the control group. The expression levels of these cytokines were elevated in the HFD mice at week eight. Values are the mean ±1 SD of five mice per group. * = P<0.05 with the Mann-Whitney U test.

Figure 3 depicts features of osteophyte formation at high magnification. The IPFP volume was slightly increased in the HFD group by week four (Figure 3d). Inflammatory features, including enhanced angiogenesis (arrows) and infiltration of macrophage-like round synovial cells (asterisk) were observed at the anterior edge of the tibial cartilage by week four in the HFD group (Figure 3d, e). Cartilaginous osteophytes gradually developed in the same region (Figure 3e, h, f, i, arrowhead), and the features of synovitis continued by twelve weeks (Figure 3f). Ossification of cartilaginous osteophytes was apparent, with size and maturity increasing from eight weeks to twelve weeks of the HFD (Figure 3f, i, arrowhead, Figure 4a). Osteophytes formed predominantly at the antero-medial region of the tibial plateau, so that the size of the osteophyte was larger in Lane 1 compared to Lane 2 or 3 (data not shown). Histological evaluation indicated the cartilage destruction score of the HFD group was significantly increased after 8 weeks of the HFD (Figure 4b, P<0.05).

To elucidate the implication of adipogenesis and angiogenesis in osteophyte formation, the correlations between histological values and osteophyte volume were evaluated. As shown in Figure 4c, osteophyte area was significantly associated with IPFP area, individual adipocyte area and vascular area. Correlation of these values (R^2^ = 0.5923 for osteophyte area and IPFP area, R^2^ = 0.6358 for osteophyte area and adipocyte area, R^2^ = 0.4376 for osteophyte area and vascular area) were strong.

### mRNA Profiles in the IPFP

We evaluated the expression levels of inflammatory cytokines in the IPFP for the HFD and control groups, since the IPFP has recently been implicated in the pathology of osteoarthritis [13], [23]. The IPFP was excised using a surgical microscope and microsurgical technique at eight and twelve weeks after initiation of the diet. Real-time RT-PCR analysis revealed the expression levels of adipokines (Leptin, Nampt, Chemerin and Lipocalin2), inflammatory cytokines (VEGF and TNF-α), and growth factor (TGF-β) were significantly elevated in the IPFP from the eighth week of the HFD. Simultaneously, the CD68 macrophage marker was increased in the IPFP from the eighth week of the HFD (Figure 4d). The expression of adiponectin, which is reported to play a protective role in OA [24], was not affected by HFD both in IPFP and serum (Figure 4d).

To elucidate the association between adipokines and inflammatory cytokines, the correlation coefficient among their expression levels were calculated. As shown in Figure 4a, adipokine expression and TNF-α, VEGF, and TGF-β were significantly correlated. In addition, CD68 expression positively associated with Nampt expression, indicating the influence of macrophages on Nampt expression.

**Figure 5:**
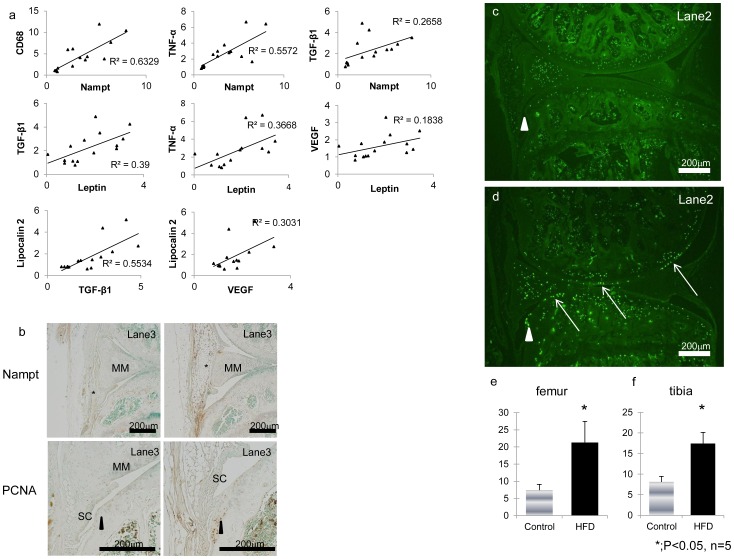
Correlation between adipokines and inflammatory cytokines or CD68 and immunohistological evaluation of knee joints. (a); Correlation between mRNA expression of adipokines, such as Leptin and mRNA expression of Nampt, and CD68 or inflammatory cytokines. The expression of Leptin and Nampt were positively correlated with TNF-α, VEGF and TGF-β, Nampt expression was strongly correlated with the macrophage marker CD68. (b); Immunostaining for Nampt and PCNA in the IPFP from week twelve of the HFD and in controls. Nampt protein expression was increased at the IPFP in the HFD. PCNA positive cells were abundantly observed at the peripheral region of osteophyte formation in the HFD group. (c, d); Analysis of apoptosis in TUNEL-stained knee joint sections from HFD and control mice. The number of TUNEL-positive cells was increased in knee joint cartilage from mice in the HFD group especially at the areas with osteophytes (triangle) and the superficial layer of articular cartilage (arrows). (e, f); The number of TUNEL-positive cells per section in the superficial layer of the articular cartilage was determined under fluorescence microscopy. Reported values are the mean ±1 SD of five mice per group. * = P<0.05 by Mann-Whitney U test.

The expression of NAMPT is increased in the plasma and synovial fluid of patients with OA [25]. Immunohistological analysis for Nampt was performed to verify the spatial and temporal expression pattern of Nampt. Immunohistological examination revealed that Nampt protein was highly expressed in the IPFP at the twelfth week of the HFD (Figure 5a, b). Of note, Nampt expression was condensed in the vicinity of osteophyte formation (Figure 5a, b).

Immunostaining for PCNA, a marker for proliferation, was conducted to estimate cell turnover activity at the site of osteophyte formation. PCNA positive cells were abundantly observed at the site of osteophyte formation in the HFD group (Figure 5d). These observations indicated an enhanced state of cell turnover at this region.

### Chondrocyte Apoptosis in HFD Models

Apoptotic cells exist abundantly at the chondro-osteophyte, which is observed at the peripheral area of osteoarthritic joints, even in the early stage of the disease [26]. Chondrocyte apoptosis is increased in OA cartilage and is anatomically linked to proteoglycan depletion [27]. These observations prompted us to investigate the effect of the HFD on chondrocyte apoptosis. TUNEL staining was performed for the lane 2 section in the HFD mice and controls at week eight of the diet. TUNEL-positive cells were abundantly observed in the superficial layer of the articular cartilage (Figure 5c) and at the site of osteophyte formation (Figure 5c, d, arrows) in the HFD mice. The number of TUNEL-positive cells in the articular cartilage was significantly increased in the HFD group (Figure 5e, f). Combined with enhanced proliferation of chondrocytes in this region, the knee joint in the HFD group is in the course of osteoarthritic change, including osteophyte formation.

## Discussion

Our findings revealed that a HFD induces hypertrophy of the IPFP in association with inflammation in less than three months time. In order to objectively quantify the severity of OA development in the HFD model, we evaluated serial sections for histological findings. We quantified the severity of OA development using osteophyte volume and the number of TUNEL-positive cells rather than scoring of the visual findings.

Several laboratories have succeeded in developing murine knee OA with more than a three-month exposure to a HFD. OA susceptibility for HFD mice depends on mice strain and gender [28]. For example, mice of strain Dba respond less readily to the HFD than mice of strain C57 black [28]. Furthermore, female mice are less susceptible to the change in dietary regimen than males. Male mice of strain C57 black fed a diet containing 29% fat from the age of six or twelve months to the end of their lives showed an accelerated onset and an increased incidence of OA as compared with control mice fed a stock diet containing 5% fat [19]. Our study reveals the initiation of OA change occurs at an earlier period than previously reported.

We demonstrated enhanced angiogenesis, infiltration of macrophage-like cells and increased synovium at the anterior edge of the tibial cartilage in advance of and in combination with osteophyte formation by week eight of the HFD. We also found enhanced adipokine secretion with IPFP hypertrophy, followed by aggregation of synovial cells. The IPFP has recently been implicated as an additional joint tissue involved in the development and progression of knee-OA [13], [23]. The human osteoarthritic knee IPFP was found to contain significantly elevated protein levels of inflammatory cytokines and adipokines [14]. These inflammatory mediators have been found in synovial fluid and have been suggested to influence cartilage and synovial metabolism [29]. Our study demonstrated that the mRNA expression levels of inflammatory cytokines, such as VEGF and TNF-α, and adipokines, such as Leptin and Nampt, and growth factors, such as TGF-β, were enhanced at week eight of the HFD which is consistent with previously reported data [14], [25], [29], [30].

We clearly exhibited adipocyte hypertrophy and increased angiogenesis were strongly correlated with osteophyte volume (Figure 3c). Furthermore, the expression of adipokines (Nampt and Leptin) and adipocyte hypertrophy markers (Lipocalin2 and Chemerin) was correlated with expression of TGF-β and inflammatory cytokines in the IPFP (Figure 4a). These results indicate adipocyte hypertrophy closely links osteophyte formation through secretion of inflammatory cytokines.

Leptin has been detected in synovial fluid (SF) obtained from patients with OA [30]. Leptin expression is also enhanced in both osteophyte and cartilage tissue obtained from patients with OA. Leptin is reported to act as a pro-inflammatory adipokine with a catabolic role in cartilage metabolism via the up regulation of proteolytic enzymes [31]. However, Leptin but not Adiponectin promoted the expression of cartilage-specific markers through mitogen-activated protein kinase, Janus kinase and phosphatidylinositol-3 kinase signaling pathways [32]. The metabolic function of Leptin may play a pivotal role in osteophyte formation considering the enhanced expression level of leptin in the IPFP.

The expression of NAMPT is increased in the plasma and synovial fluid of patients with OA [25]. Although NAMPT has been reported to be produced by chondrocytes from OA patients, our study has demonstrated the highly enhanced expression of Nampt in the IPFP in response to a HFD. Nampt production is increased by IL-1β in chondrocytes [33]. Moreover, Nampt induces PGE2 release in articular chondrocytes as a result of increased mPGES-1 and decreased 15-PGDH synthesis [33]. NAMPT also triggers the synthesis and release of MMP-3, MMP-13, ADAMTS-4, and ADAMTS-5 by chondrocytes [33]. Thus, Nampt may play a pivotal role in chondrocyte metabolism, including osteophyte formation. The mechanism for regulation of Nampt expression in the IPFP has yet to be discovered.

### 

#### Adipose tissue macrophage infiltration during obesity

We observed that the CD68 macrophage marker was increased in the IPFP at week eight of the HFD and that this occurred simultaneously with the enhancement of Nampt and TNF-α expression. Nampt mRNA level is strongly correlated with the CD68 macrophage-specific marker and TNF-α mRNA levels in adipose tissues [34]. In this study, we report a strong correlation between mRNA expression of Nampt and CD68 in the IPFP (Figure 4a). TNF-α is a pro-inflammatory cytokine produced mainly by macrophages and lymphocytes. It is also produced by adipose tissue although the expression level is low in humans [35]. Large-scale studies of gene expression using micro-array approaches have revealed that variations in gene expression in white adipose tissues (WAT) are essentially related to a macrophage infiltration in WAT of obese mice [10]. Thus, locally present CD68-positive macrophages may play important roles in the augmentation of these cytokines. Also, studies have shown an increased inflammatory response associated with the presence of hyperleptinemia without obesity [36], [37], and that leptin is able to control TNF-α production and activation by macrophages [36]. We have shown TNF-α expression to be significantly associated with Nampt and Leptin expression (Figure 4a). These observations suggest Leptin may also regulate TNF-α expression in the IPFP.

The expression of Chemerin and Lipocalin2 was enhanced in the IPFP by HFD (Figure 4c). Chemerin is predominantly expressed in adipocytes and promotes calcium mobilization and chemotaxis of immature dendritic cells and macrophages [38] In 3T3-L1 adipocytes, Chemerin expression is enhanced during differentiation [*39*]. Human plasma levels of Chemerin were significantly associated with body mass index, circulating triglycerides, and blood pressure [39], indicating a potent function of Chemerin in immune and metabolic homeostasis. Lipocalin2 is a 25 kDa glycoprotein expressed in neutrophil granules, adipocytes and chondrocytes [40]–[42]. Lipocalin2 belongs to the large family of Lipocalins which have high affinity for small hydrophobic ligands such as steroids and LPS [42]. Lipocalin2 expression was dramatically enhanced in adipocytes by IL-1β treatment [43]. In the analysis of cartilage degradation and protein release using proteomics, Lipocalin2 is identified as a biomarker of cartilage degradation [44] Overexpression of Lipocalin2 in chondrocytes caused reduction in proliferation and promotion of hypertrophy [41]. These observations suggest potent roles of Chemerin and Lipocalin2 in the inflammatory responses of the IPFP and enhanced chondrocyte apoptosis in the HFD group.

On the other hand, Adiponectin levels in plasma and the IPFP were not affected by HFD in our study. Adiponectin has been shown to be implicated in the pathogenesis of osteoarthritis [42]. Plasma Adiponectin levels were reported to be significantly higher in patients with OA than in healthy controls [45]. Conversely, Adiponectin concentrations in plasma and synovial fluid show significant inverse correlation with disease severity, suggesting a possible protective role of Adiponectin in OA [24]. Perhaps an extended time course of the HFD may alter Adiponectin levels in the IPFP.

We showed that TGF-β mRNA expression was gradually enhanced by a HFD. The ability of TGF-β to induce osteophyte formation was previously demonstrated [46], [47]. There is significant overlap in the location of TGF-β-induced and experimental OA–induced osteophyte formation [46]. These observations confirm that TGF-β plays a role in osteophyte development during experimental OA [48]. Leptin, which is up regulated in parallel with TGF-β in this study, stimulates chondrocyte synthesis of TGF-β in animal experiments [30]. Thus, Leptin may act as a trigger to stimulate TGF-β expression from the IPFP. Consistently, we observed TGF-β mRNA expression was positively correlated with Leptin expression in the IPFP (Figure 4a).

It is still unclear whether the events observed in the IPFP are directly induced by HFD or are an indirect response to the destruction of articular cartilage. For instance, gain of weight in HFD mice may result in an increase in mechanical load and trigger the wear of cartilage, followed by local inflammation. To elucidate this problem, further experiments will be required.

In conclusion, we have shown that articular cartilage degradation and osteophyte formation can be triggered from as early as eight weeks of a HFD with detailed evaluation methods. Our observations suggest pivotal roles for the IPFP in the development of osteophyte formation and cartilage degradation. Furthermore, our methods gave the ability to adjust and reproduce the episodes of applied mechanical loading and metabolic alteration. This provided an opportunity to investigate articular cartilage responses to metabolic stress and the mechanisms involved in the progression of OA.

## References

[1] Kamekura S, Hoshi K, Shimoaka T, Chung U, Chikuda H, et al (2005) Osteoarthritis development in novel experimental mouse models induced by knee joint instability. Osteoarthritis Cartilage 13: 632–641. 15896985 10.1016/j.joca.2005.03.004

[2] Gabay O, Hall DJ, Berenbaum F, Henrotin Y, Sanchez C (2008) Osteoarthritis and obesity: experimental models. Joint Bone Spine. France. 675–679. 10.1016/j.jbspin.2008.07.011 PMC2768592 19022697

[3] Anderson JJ, Felson DT (1988) Factors associated with osteoarthritis of the knee in the first national Health and Nutrition Examination Survey (HANES I). Evidence for an association with overweight, race, and physical demands of work. Am J Epidemiol 128: 179–189. 3381825 10.1093/oxfordjournals.aje.a114939

[4] Magliano M (2008) Obesity and arthritis. Menopause Int. England. 149–154. 10.1258/mi.2008.008018 19037063

[5] Hashimoto S, Ochs RL, Komiya S, Lotz M (1998) Linkage of chondrocyte apoptosis and cartilage degradation in human osteoarthritis. Arthritis & Rheumatism 41: 1632–1638. 9751096 10.1002/1529-0131(199809)41:9<1632::AID-ART14>3.0.CO;2-A

[6] Andriacchi TP, Mündermann A, Smith RL, Alexander EJ, Dyrby CO, et al. (2004) A Framework for the *in Vivo* Pathomechanics of Osteoarthritis at the Knee. 447–457. 10.1023/b:abme.0000017541.82498.37 15095819

[7] Carman WJ, Sowers M, Hawthorne VM, Weissfeld LA (1994) Obesity as a risk factor for osteoarthritis of the hand and wrist: a prospective study. Am J Epidemiol 139: 119–129. 8296779 10.1093/oxfordjournals.aje.a116974

[8] Dahaghin S, Bierma-Zeinstra SM, Koes BW, Hazes JM, Pols HA (2007) Do metabolic factors add to the effect of overweight on hand osteoarthritis? The Rotterdam Study. Ann Rheum Dis. England. 916–920. 10.1136/ard.2005.045724 PMC1955104 17314121

[9] Kershaw EE, Flier JS (2004) Adipose tissue as an endocrine organ. J Clin Endocrinol Metab. United States. 2548–2556. 10.1210/jc.2004-0395 15181022

[10] Weisberg SP, McCann D, Desai M, Rosenbaum M, Leibel RL, et al (2003) Obesity is associated with macrophage accumulation in adipose tissue. The Journal of Clinical Investigation 112: 1796–1808. 14679176 10.1172/JCI19246 PMC296995

[11] Odegaard JI, Chawla A (2008) Mechanisms of macrophage activation in obesity-induced insulin resistance. Nat Clin Pract Endocrinol Metab. England. 619–626. 10.1038/ncpendmet0976 PMC3381907 18838972

[12] Hotamisligil GS, Erbay E (2008) Nutrient sensing and inflammation in metabolic diseases. Nat Rev Immunol 8: 923–934. 19029988 10.1038/nri2449 PMC2814543

[13] Clockaerts S, Bastiaansen-Jenniskens YM, Runhaar J, Van Osch GJ, Van Offel JF, et al (2010) The infrapatellar fat pad should be considered as an active osteoarthritic joint tissue: a narrative review. Osteoarthritis Cartilage 18: 876–882. 20417297 10.1016/j.joca.2010.03.014

[14] Ushiyama T, Chano T, Inoue K, Matsusue Y (2003) Cytokine production in the infrapatellar fat pad: another source of cytokines in knee synovial fluids. Ann Rheum Dis 62: 108–112. 12525378 10.1136/ard.62.2.108 PMC1754438

[15] Griffin TM, Fermor B, Huebner JL, Kraus VB, Rodriguiz RM, et al (2010) Diet-induced obesity differentially regulates behavioral, biomechanical, and molecular risk factors for osteoarthritis in mice. Arthritis Res Ther 12: R130. 20604941 10.1186/ar3068 PMC2945020

[16] Wilhelmi G, Faust R (1976) Suitability of the C57 black mouse as an experimental animal for the study of skeletal changes due to ageing, with special reference to osteo-arthrosis and its response to tribenoside. Pharmacology 14: 289–296. 947093 10.1159/000136607

[17] Säämänen AMK, Salminen HJ, Dean PB, De Crombrugghe B, Vuorio EI, et al (2000) Osteoarthritis-like lesions in transgenic mice harboring a small deletion mutation in type II collagen gene. Osteoarthritis and Cartilage 8: 248–257. 10903878 10.1053/joca.2000.0298

[18] Walton M (1979) Patella displacement and osteoarthrosis of the knee joint in mice. J Pathol 127: 165–172. 469642 10.1002/path.1711270402

[19] Silberberg M, Silberberg R (1952) Degenerative joint disease in mice fed a high-fat diet at various ages. Exp Med Surg 10: 76–87. 14954894

[20] Sokoloff L, Mickelsen O, Silverstein E, Jay GE Jr, Yamamoto RS (1960) Experimental obesity and osteoarthritis. Am J Physiol 198: 765–770. 13832538 10.1152/ajplegacy.1960.198.4.765

[21] Griffin TM, Huebner JL, Kraus VB, Yan Z, Guilak F (2012) Induction of osteoarthritis and metabolic inflammation by a very high-fat diet in mice: effects of short-term exercise. Arthritis Rheum 64: 443–453. 21953366 10.1002/art.33332 PMC3268860

[22] Tozuka Y, Wada E, Wada K (2009) Diet-induced obesity in female mice leads to peroxidized lipid accumulations and impairment of hippocampal neurogenesis during the early life of their offspring. FASEB J 23: 1920–1934. 19158155 10.1096/fj.08-124784

[23] Distel E, Cadoudal T, Durant S, Poignard A, Chevalier X, et al (2009) The infrapatellar fat pad in knee osteoarthritis: an important source of interleukin-6 and its soluble receptor. Arthritis Rheum 60: 3374–3377. 19877065 10.1002/art.24881

[24] Honsawek S, Chayanupatkul M (2010) Correlation of plasma and synovial fluid adiponectin with knee osteoarthritis severity. Arch Med Res. United States: A 2010 IMSS. Published by Elsevier Inc. 593–598. 10.1016/j.arcmed.2010.11.007 21199727

[25] Presle N, Pottie P, Dumond H, Guillaume C, Lapicque F, et al (2006) Differential distribution of adipokines between serum and synovial fluid in patients with osteoarthritis. Contribution of joint tissues to their articular production. Osteoarthritis Cartilage 14: 690–695. 16527497 10.1016/j.joca.2006.01.009

[26] Doi T, Nishida K, Matsuo M, Yoshida A, Murakami T, et al.. (2002) Evidence of oncotic cell death and DNA fragmentation in human hypertrophic chondrocytes in chondro-osteophyte. Osteoarthritis Cartilage. England: 2002 OsteoArthritis Research Society International. 270–276. 10.1053/joca.2001.0503 11950249

[27] Blanco F, Guitian R, Vázquez-Martul E, de Toro F, Galdo F (1998) Osteoarthritis chondrocytes die by apoptosis. A possible pathway for osteoarthritis pathology. Arthritis Rheum 41: 284–289. 9485086 10.1002/1529-0131(199802)41:2<284::AID-ART12>3.0.CO;2-T

[28] Silberberg M, Silberberg R (1951) Skeletal growth, aging and osteoarthritis; effects of enriched diets and of ovariectomy. Bull Hosp Joint Dis 12: 256–272. 14905109

[29] Schaffler A, Ehling A, Neumann E, Herfarth H, Tarner I, et al.. (2003) Adipocytokines in synovial fluid. JAMA. United States. 1709–1710. 10.1001/jama.290.13.1709-c 14519703

[30] Dumond H, Presle N, Terlain B, Mainard D, Loeuille D, et al (2003) Evidence for a key role of leptin in osteoarthritis. Arthritis & Rheumatism 48: 3118–3129. 14613274 10.1002/art.11303

[31] Hui W, Litherland GJ, Elias MS, Kitson GI, Cawston TE, et al.. (2011) Leptin produced by joint white adipose tissue induces cartilage degradation via upregulation and activation of matrix metalloproteinases. Ann Rheum Dis. 10.1136/annrheumdis-2011-200372 22072016

[32] Francin PJ, Guillaume C, Humbert AC, Pottie P, Netter P, et al (2011) Association between the chondrocyte phenotype and the expression of adipokines and their receptors: evidence for a role of leptin but not adiponectin in the expression of cartilage-specific markers. J Cell Physiol 226: 2790–2797. 21935928 10.1002/jcp.22627

[33] Gosset M, Berenbaum F, Salvat C, Sautet A, Pigenet A, et al (2008) Crucial role of visfatin/pre-B cell colony-enhancing factor in matrix degradation and prostaglandin E2 synthesis in chondrocytes: possible influence on osteoarthritis. Arthritis Rheum 58: 1399–1409. 18438860 10.1002/art.23431

[34] Chang YC, Chang TJ, Lee WJ, Chuang LM (2010) The relationship of visfatin/pre-B-cell colony-enhancing factor/nicotinamide phosphoribosyltransferase in adipose tissue with inflammation, insulin resistance, and plasma lipids. Metabolism 59: 93–99. 19765775 10.1016/j.metabol.2009.07.011

[35] Bastard JP, Maachi M, Lagathu C, Kim MJ, Caron M, et al (2006) Recent advances in the relationship between obesity, inflammation, and insulin resistance. Eur Cytokine Netw 17: 4–12. 16613757

[36] Loffreda S, Yang SQ, Lin HZ, Karp CL, Brengman ML, et al (1998) Leptin regulates proinflammatory immune responses. FASEB J 12: 57–65. 9438411

[37] van Dielen FM, van't Veer C, Schols AM, Soeters PB, Buurman WA, et al (2001) Increased leptin concentrations correlate with increased concentrations of inflammatory markers in morbidly obese individuals. Int J Obes Relat Metab Disord 25: 1759–1766. 11781755 10.1038/sj.ijo.0801825

[38] Wittamer V, Franssen JD, Vulcano M, Mirjolet JF, Le Poul E, et al.. (2003) Specific recruitment of antigen-presenting cells by chemerin, a novel processed ligand from human inflammatory fluids. J Exp Med. United States. 977–985. 10.1084/jem.20030382 PMC2194212 14530373

[39] Bozaoglu K, Bolton K, McMillan J, Zimmet P, Jowett J, et al.. (2007) Chemerin is a novel adipokine associated with obesity and metabolic syndrome. Endocrinology. United States. 4687–4694. 10.1210/en.2007-0175 17640997

[40] Triebel S, Blaser J, Reinke H, Tschesche H (1992) A 25 kDa alpha 2-microglobulin-related protein is a component of the 125 kDa form of human gelatinase. FEBS Lett. Netherlands. 386–388. 10.1016/0014-5793(92)81511-j 1281792

[41] Owen HC, Roberts SJ, Ahmed SF, Farquharson C (2008) Dexamethasone-induced expression of the glucocorticoid response gene lipocalin 2 in chondrocytes. Am J Physiol Endocrinol Metab. United States. E1023–1034. 10.1152/ajpendo.00586.2007 18381927

[42] Gomez R, Conde J, Scotece M, Gomez-Reino JJ, Lago F, et al.. (2011) What's new in our understanding of the role of adipokines in rheumatic diseases? Nat Rev Rheumatol. United States. 528–536. 10.1038/nrrheum.2011.107 21808287

[43] Sommer G, Weise S, Kralisch S, Lossner U, Bluher M, et al (2009) Lipocalin-2 is induced by interleukin-1beta in murine adipocytes in vitro. J Cell Biochem 106: 103–108. 19009554 10.1002/jcb.21980

[44] Wilson R, Belluoccio D, Little CB, Fosang AJ, Bateman JF (2008) Proteomic characterization of mouse cartilage degradation in vitro. Arthritis Rheum 58: 3120–3131. 18821673 10.1002/art.23789

[45] Laurberg TB, Frystyk J, Ellingsen T, Hansen IT, Jorgensen A, et al.. (2009) Plasma adiponectin in patients with active, early, and chronic rheumatoid arthritis who are steroid- and disease-modifying antirheumatic drug-naive compared with patients with osteoarthritis and controls. J Rheumatol. Canada. 1885–1891. 10.3899/jrheum.080907 19684150

[46] Blaney Davidson EN, Vitters EL, van Beuningen HM, van de Loo FA, van den Berg WB, et al (2007) Resemblance of osteophytes in experimental osteoarthritis to transforming growth factor beta-induced osteophytes: limited role of bone morphogenetic protein in early osteoarthritic osteophyte formation. Arthritis Rheum 56: 4065–4073. 18050218 10.1002/art.23034

[47] van der Kraan PM, van den Berg WB (2007) Osteophytes: relevance and biology. Osteoarthritis Cartilage 15: 237–244. 17204437 10.1016/j.joca.2006.11.006

[48] Scharstuhl A, Glansbeek HL, van Beuningen HM, Vitters EL, van der Kraan PM, et al (2002) Inhibition of endogenous TGF-beta during experimental osteoarthritis prevents osteophyte formation and impairs cartilage repair. J Immunol 169: 507–514. 12077282 10.4049/jimmunol.169.1.507

